# Transcriptomic profiles of the maternal-to-zygotic transition for *Drosophila* embryos exposed to acute and chronic heat stress

**DOI:** 10.1093/g3journal/jkag203

**Published:** 2026-08-13

**Authors:** Faizan Rashid, Natalie Biel, Jenny Drnevich, Anna Marie Sokac

**Affiliations:** Department of Cell and Developmental Biology, University of Illinois Urbana-Champaign, Urbana, IL 61801, United States; Department of Cell and Developmental Biology, University of Illinois Urbana-Champaign, Urbana, IL 61801, United States; Integrative Molecular and Biomedical Sciences Program, Baylor College of Medicine, Houston, TX 77030, United States; Roy J. Carver Biotechnology Center, University of Illinois Urbana-Champaign, Urbana, IL 61801, United States; Department of Cell and Developmental Biology, University of Illinois Urbana-Champaign, Urbana, IL 61801, United States

**Keywords:** *Drosophila*, RNA sequencing (RNA-seq), embryo, maternal-to-zygotic transition (MZT), zygotic genome activation, heat shock response (HSR)

## Abstract

Heat stress challenges embryo survival, but the molecular reasons for this are unclear. We investigated how heat stress alters the maternal-to-zygotic transition (MZT) during *Drosophila melanogaster* development. Using RNA sequencing, we characterized the MZT under nonstress, acute, and chronic heat stress conditions. MZT genes were defined as those differentially expressed between the minor and major waves of zygotic genome activation. MZT genes were associated with multiple processes, including transcription, splicing, translation, and development. Under acute stress, the MZT deviated little from nonstressed embryos except that heat shock protein (HSP) genes were activated, and maternal transcript clearance was minimally misregulated. Under chronic stress, a core MZT persisted; but stress-related gene expression was obvious, maternal transcripts were strongly misregulated, and embryos showed poor survival. Overall, the MZT is robust, but increasingly falters with more severe stress. We present our dataset as a resource to aid molecular understanding of embryo resilience and vulnerability to environmental stressors.

## Introduction

Heat stress adversely impacts the development of diverse organisms (Graham et al. 1998; Puscheck et al. 2015; Zhu et al. 2021; Lang et al. 2024; Gibbs et al. 2025). In mammalian embryos, heat stress can disrupt molecular processes in the nucleus and cytoplasm, ultimately compromising organismal structure, function, and viability (Ozawa et al. 2002; Paula-Lopes and Hansen 2002; Matsuzuka et al. 2005; Moretti et al. 2005; de Barros and Paula-Lopes 2018; Camargo et al. 2019; Feng et al. 2024; Trigg et al. 2024; Held-Hoelker et al. 2025). Similarly, in embryos of the fruit fly *Drosophila*, heat stress disrupts transcription, chromatin remodeling, translation, cell cycle fidelity, and developmental timing, leading to morphogenesis defects and reduced larval hatching (Lindquist 1981; Maroto and Sierra 1988; Vazquez et al. 1993; Welte et al. 1993; Zheng et al. 2013; Kuntz and Eisen 2014; Crews et al. 2016; Lockwood et al. 2017; Chong et al. 2018; Figard et al. 2019; Crapse et al. 2021; Kale et al. 2026; Örkenby et al. 2023; Mikucki et al. 2024; Gibbs et al. 2025; O'Leary et al. 2025). In both mammals and *Drosophila*, heat stress is uniquely harmful to early embryos, while later embryos show higher heat tolerance (Graziosi et al. 1980; Welte et al. 1993; Hansen 2009; Lockwood et al. 2018; Borda et al. 2021). Understanding of this early life sensitivity is lacking.

One outstanding question is whether early embryos can mount an inducible stress response while simultaneously coordinating all events required for successful development (Hamdoun and Epel 2007; Lockwood et al. 2017; Mikucki et al. 2024; O'Leary et al. 2025). For example, inducible heat shock response (HSR) demands transcriptional upregulation of genes encoding protective heat shock proteins (HSPs) and accompanying downregulation of genes unrelated to stress (Pessa et al. 2024). But is such transcriptional remodeling compatible with the precise gene expression programs needed to support early embryo patterning and morphogenesis? Furthermore, some molecular players required for HSR are alternately needed for normal developmental functions (Åkerfelt et al. 2010). Both HSPs, themselves, and their activating transcription factor, heat shock factor-1 (HSF1), contribute to microtubule organization, protein and mRNA localization, and early embryo survival (Christians et al. 2000; Basto et al. 2007; Song et al. 2007; Palumbo et al. 2020), so shifting HSP or HSF1 activity toward stress response may compromise rather than safeguard embryos. Finally, high levels of HSPs, such as Hsp70, can exert their own detrimental impacts on cellular processes critical to development, including cell division (Welte et al. 1993; Wang and Lindquist 1998). Thus, interactions between heat stress response and embryogenesis appear to be complex and consequential for both the form and viability of the future organism (Krebs and Feder 1998; Rutherford and Lindquist 1998; Nollen and Morimoto 2002; Hamdoun and Epel 2007; Mikucki et al. 2024; Pessa et al. 2024).

Here, we asked how heat stress interacts with the conserved regulatory switch in early embryos known as the maternal-to-zygotic transition (MZT) (Vastenhouw et al. 2019; Brantley and Di Talia 2024; Kojima et al. 2025). After fertilization, embryos first rely on maternally contributed mRNAs and proteins to support their initial development. At the MZT, much of this maternal contribution is degraded and replaced by new gene products transcribed from the zygote's own genome. Thus, the MZT represents the necessary handing over of developmental control from mother to zygote. Many cellular processes accompany the MZT, including chromatin remodeling, transcription, RNA processing, translation, RNA and protein degradation, and cell cycle and metabolic remodeling (Blythe and Wieschaus 2016; Hamm and Harrison 2018; Riemondy et al. 2023; Brantley and Di Talia 2024; Kojima et al. 2025; Pérez-Mojica et al. 2025); and any of these processes could be modified by heat stress response. We were specifically interested to learn how gene expression from the zygotic genome changes during the MZT in heat-stressed embryos.

We generated our dataset by RNA sequencing (RNA-seq), comparing transcriptomes between control nonstressed and heat-stressed *D. melanogaster* embryos. We found that HSR is induced by both acute and chronic heat stress, as evidenced by the upregulation of HSP genes. This stress response did not halt the MZT, as expected gene expression changes were still observed following both acute and chronic heat stress. However, disruption of the MZT increased with more severe stress and correlated with reduced embryo survival. This work is relevant to developmental biologists with interest in the MZT, and ecologists and reproductive biologists with interest in embryo fitness and health, particularly in the context of environmental stress.

## Materials and methods

### Experimental design

Upon fertilization, the early *D. melanogaster* embryo undergoes 13 rapid and synchronous nuclear division cycles (NCs) without cytokinesis to generate a syncytial embryo. At NC14, the embryo completes cellularization to convert the syncytium into a cellular embryo (Sokac et al. 2023). During NCs 1 to 8, development depends on maternally deposited mRNAs and proteins. But with the MZT, a minor wave of zygotic genome activation begins at NC8, followed by a major wave at NC14, with the expression of tens and then thousands of genes, respectively (De Renzis et al. 2007; Lécuyer et al. 2007; Tadros and Lipshitz 2009).

To examine differentially expressed genes (DEGs) during the MZT under nonstress and heat stress conditions, we hand selected embryos early in the minor wave at NC9 (Bownes stage 3) and in the major wave at NC14 (Bownes stage 5; Fig. 1a). These NCs were chosen due to the unambiguous morphological hallmarks observed at these cycles (Campos-Ortega and Hartenstein 1985). Specifically, at NC9, which starts ∼10 min after the onset of the minor wave, pole cells first bulge from the posterior end of the embryo; while at NC14, a furrow front moves uniformly from embryo periphery to interior as cellularization progresses. These observables are readily detected under a dissecting microscope.

**Fig. 1.**
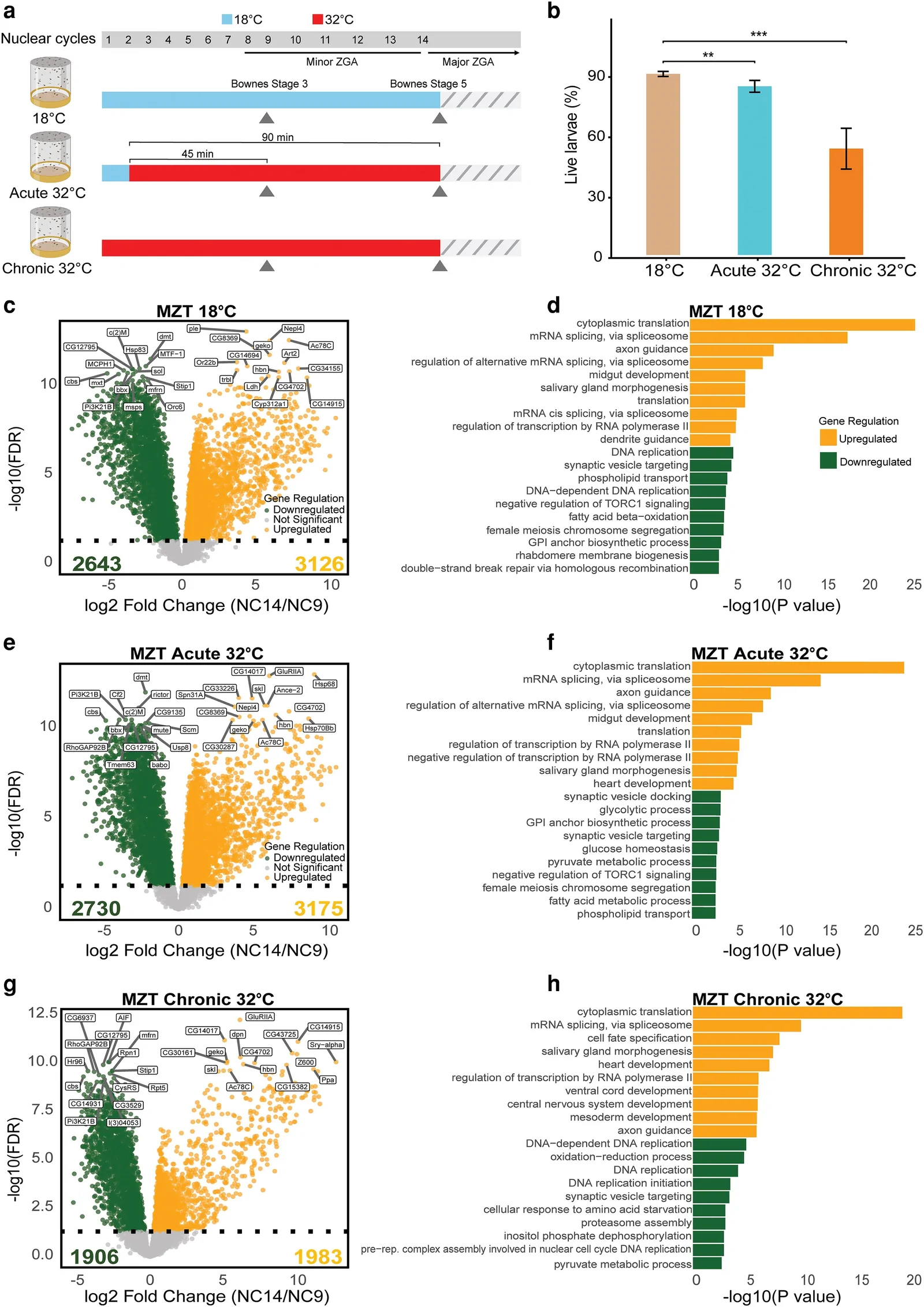
DGE during the MZT for nonstress control and heat-stressed embryos. a) Schematic showing embryo collection schemes. Wild-type *D. melanogaster* embryos were hand selected at NC9 and NC14 (indicated by arrowheads at Bownes stages 3 and 5, respectively) for each condition (nonstress 18 °C control, acute 32 °C heat stress, and chronic 32 °C heat stress), followed by RNA-seq (ZGA, zygotic genome activation). Hatched segment of experimental scheme indicates continued embryo development for the larval hatching assays. Images of embryo collection cups created in BioRender. b) Bar plots showing percentage (%) of embryos that hatched as live larvae for each condition (nonstress 18 °C control, acute 32 °C heat stress, and chronic 32 °C heat stress; n ≥ 600 embryos per condition, pooled from 3 biological replicates). Significance thresholds were set at *P* ≤ 0.01 (**) and *P* ≤ 0.001 (***). c, e, and g) Volcano plots showing differential expression between NC9 and NC14 embryos under each condition (orange, upper right wing = upregulated; green, upper left wing = downregulated; *x* axes show logFC, and *y* axes show −log_10_(FDR). The top 15 upregulated and downregulated genes are labeled. d, f, and h) GO Biological Process enrichment (top 10 terms ranked by −log_10_(*P* value)) for upregulated (orange, top bars) and downregulated (green, bottom bars) gene sets. Abbreviation: pre-rep., pre-replicative.

Regarding experimental temperatures, *D. melanogaster* embryos can develop successfully across a 20 °C or more temperature range (7 to 35 °C based on extremes from the literature), with peak viability maintained from 15 to 27 °C (Kuntz and Eisen 2014; Chen et al. 2015; Schou et al. 2017; Chong et al. 2018; Crapse et al. 2021; Falahati et al. 2021; Kale et al. 2026; Gibbs et al. 2025; O'Leary et al. 2025). We chose 18 °C as the control nonstress temperature for several reasons: First, 18 °C is well within the confines of temperatures supporting highest larval hatching rates (Schou et al. 2017). Second, 18 °C has been described as the temperature of maximum gene expression canalization between wild-type *Drosophila* strains (Chen et al. 2015). Third, our previous work on the actin cytoskeleton showed that 18 °C is an optimal temperature for ensuring the success of morphogenesis across genotypic perturbations (Zheng et al. 2013; Figard et al. 2019; Biel et al. 2020). We chose 32 °C as an intermediate heat stress temperature because the literature consistently reports that sustained growth at ≥30 °C is stressful while ≥35 °C is lethal (Kuntz and Eisen 2014; Schou et al. 2017; Chong et al. 2018; Crapse et al. 2021; Falahati et al. 2021; Gibbs et al. 2025).

To test whether an increase in the duration of 32 °C heat stress has a consequent increase in impact on the MZT, we compared an acute versus a chronic heat stress condition (Fig. 1a). The acute 32 °C heat stress was applied either for 45 min for embryos collected for the NC9 timepoint or for 90 min for embryos collected for the NC14 timepoint. Specifically, this means that heat stress was applied 45 min prior to the NC9 timepoint, such that within 90 min, embryos reach NC14 (see Fig. 1a). The duration of heat exposure in the acute stress condition was dictated solely by the developmentally defined time windows between egg laying and NC9 and between NC9 and NC14. The chronic stress was applied to mothers for 24 h such that resulting embryos had experienced heat exposure even as oocytes, prior to egg activation and fertilization (Avilés-Pagán and Orr-Weaver 2018). Once laid, these embryos were continually reared at 32 °C until they were collected either for the NC9 or NC14 timepoints. Larval hatching assays showed that viability was reduced to a greater extent with increasing duration of heat stress (Fig. 1b), suggesting that our acute and chronic conditions represent a moderate and more severe heat stress, respectively. Overall, this experimental design allowed us to assess how varying intensities of heat stress affect gene expression during the MZT.

### *Drosophila* husbandry, heat exposure and embryo collection

The inbred wild-type *D. melanogaster* stock, Oregon-R (Ore-R), was maintained on Fly Food R purchased from Lab-Express, Ann Arbor, MI, USA. For embryo collection, adult flies (3 to 5 d old) were placed in embryo collection cups with apple juice agar plates with a dab of active yeast paste (Figard and Sokac 2011). Per cup ∼100 virgin females were crossed with ∼50 males.

Because developmental timing and events scale with temperature (i.e. development progresses faster at increasing temperature as in Kuntz and Eisen (2014), Crapse et al. (2021), and Falahati et al. (2021)), we used visible morphology to stage all embryos. Embryos were hand selected at NC9 and NC14 as follows: NC9 embryos (Bownes stage 3) were selected based on the initial appearance of pole cells budding from the posterior of the embryo and minimal peripheral cytoplasmic clearing. NC14 embryos (Bownes stage 5) were selected based on maximum peripheral cytoplasmic clearing, observation of a cellularization furrow front (which can be seen ∼30 min after the onset of NC14), and lack of any gastrulation movements (Bownes 1975; Campos-Ortega and Hartenstein 1985). These criteria limit the selection window to <10 and <20 min for NC9 and NC14, respectively. For each plate at the indicated NC and stress condition, 5 to 10 embryos were selected and snap frozen together in liquid nitrogen and stored at −80 °C for later RNA extraction.

For the nonstress 18 °C control, cups were incubated at 18 °C until vigorous egg laying started (48 h). At this point, a fresh plate was applied to the cup at 18 °C and harvested ∼1 h later. This plate, with mixed stage embryos, was incubated at 18 °C for either 100 min before morphological selection of NC9 embryos or 4 h before selection of NC14 embryos.

For acute 32 °C heat stress, cups were incubated at 18 °C until vigorous egg laying started (48 h). At this point, a fresh plate was applied to the cup at 18 °C and harvested ∼1 h later. But then this plate, with mixed stage embryos, was switched to incubation at 32 °C for either 45 min before morphological selection of NC9 embryos or 90 min before selection of NC14 embryos. The difference in times before embryo collections at 18 °C versus 32 °C reflects the faster development of embryos as temperature increases. For example, embryos reach NC14 in ∼4 h at 18 °C, but in <2 h at 32 °C.

For chronic 32 °C heat stress, cups were incubated at 32 °C until vigorous egg laying started (24 h). This 24 h incubation provided the initial window of chronic heat stress. A fresh plate was applied to the cup at 32 °C and harvested ∼1 h later. This plate, with mixed stage embryos, was incubated at 32 °C for either 45 min before morphological selection of NC9 embryos or 90 min before selection of NC14 embryos.

### Larval hatching assays

Collection cups and plates were treated as described above for the selection of NC14 embryos for nonstress 18 °C control, acute 32 °C heat stress, or chronic 32 °C heat stress conditions. Immediately following each treatment, embryos were quickly dislodged from plates with distilled water, collected in a cell strainer, rinsed with more distilled water, and then transferred to a fresh apple juice agar plate. NC14 embryos were hand selected and aligned on another fresh apple juice agar plate. This plate was incubated in a plastic box with damp paper towels at 22 °C. After 48 h, hatching was scored as either successful if an eggshell was found empty or failed if the eggshell contained a dead embryo. Statistical analysis for hatching data was done using the χ2-contingency test in GraphPad QuickCalcs (GraphPad, San Diego, CA, USA).

### RNA extraction and sequencing

RNA extraction was done from 6 biological replicates per experimental condition, with 5 to 10 embryos each for NC9 and NC14. RNA was extracted using the RNeasy Plus Micro Kit (Catalog#74034, Qiagen, Germantown, USA) and submitted to the DNA Services Laboratory at the Roy J. Carver Biotechnology Center, University of Illinois Urbana-Champaign. RNA integrity was assayed by measuring the 28S/18S rRNA ratio and RNA quality number of purified DNase-treated total RNAs using AATI Fragment Analyzer (Advanced Analytics, Ames, USA). Stranded RNA-seq libraries were prepared using the KAPA mRNA HyperPrep Kit (Roche, Indianapolis, IN, USA). Total RNA (200 ng/sample) was enriched for polyadenylated RNA, which was then fragmented by incubation at 94 °C for 4 min. SuperScript II reverse transcriptase (Invitrogen, Thermo Fisher Scientific) and random hexamer primers were used to create first-strand cDNA. The cDNA was converted to double-stranded DNA, A-tailed at the 3′ end, blunt ended, and ligated to universal adapters. Libraries were amplified for 12 PCR cycles using Twist dual-index primers (Twist Bioscience, San Francisco, CA, USA) and then diluted to 5 nM. Library sequencing was done to produce 150 bp paired-end reads using an SP flow cell on the Illumina NovaSeq 6000 platform (Illumina, San Diego, CA, USA). Adaptor-trimmed and demultiplexed fastq read files were converted from .bcl files with bcl2fastq software (v2.2.0; Illumina, CA, USA). Read quality was ensured using FastQC (v0.11.8) analysis.

### RNA-seq analysis

#### Alignment

Reads from RNA-seq were quasi-mapped to the *D. melanogaster* transcriptome, and transcript abundances were quantified using Salmon (version 1.5.2). Transcriptome (dmel-all-transcript-r6.45.fasta.gz) and genome (dmel-all-chromosome-r6.45.fasta.gz) files were obtained from FlyBase (Öztürk-Çolak et al. 2024) to build the Salmon index using the decoy-aware method (Patro et al. 2017). To correct for sequence-specific and GC-content-related biases, seqBias and gcBias options were included. Transcript abundance estimates were computed using numBootstraps = 30. To further improve mapping precision, the validateMappings flag was enabled.

#### Estimation of gene counts

Using R (v4.4.0), gene-level counts were obtained from transcript-level quantifications with the dmel-all-r6.45.gtf file and “lengthScaledTPM” method in the tximport package (v1.34.0), which improves accuracy by accounting for transcript length and retaining multimapped reads (Soneson et al. 2016).

#### Filtering and normalization

Initial quality control checking using multidimensional scaling (MDS) clustering identified 1 chronic stress replicate from NC9 (R6_32C_ORE_S3) and 1 chronic stress replicate from NC14 (R2_32C_ORE_S5) as outliers, so they were removed. The final analysis sample sizes were 6 replicates for all conditions except these 2 where the sample size was 5 replicates. The 34 samples were filtered to exclude genes with low expression, specifically removing any genes not expressed at more than 1 count per million (CPM) in at least 5 samples. After this filtering step, 7,613 genes remained for downstream analysis. Expression data for these genes was normalized in the edgeR (v4.4.1) package using the trimmed mean of M-values (TMM) method and transformed to log_2_ counts per million with prior count = 2 (hereafter log_2_CPM). We note that the MZT involves major transcriptional changes (NC9 versus NC14) and that we violate the recommendation that TMM normalization should only be applied when a majority of genes are not differentially expressed between sample pairs (Robinson and Oshlack 2010). However, we are not aware of a better normalization method that can be applied after the fact. To allay some concern regarding the use of TMM for the MZT, we inspected the TMM values for the samples and found only small deviations from 1 (Supplementary Fig. 1), indicating that while a majority of genes may be changing, they are fairly evenly distributed between up- and downregulation. For the within NC9 or within NC14 comparisons, the use of TMM normalization is appropriate because less than half of genes are differentially expressed.

#### Differential gene expression analysis

Differential gene expression (DGE) analysis was conducted using the limma method (Law et al. 2014, 2016) on log_2_CPM, and the limma-trend pipeline was used specifically because the ratio of the largest to the smallest library size was less than 3-fold. This approach models the mean–variance relationship characteristic of RNA-seq data and applies empirical Bayes moderation to control for variance estimation across genes, which improves statistical inference when experiments have modest replicate numbers and multiple comparisons are planned (Soneson and Delorenzi 2013; Law et al. 2014, 2016; Ritchie et al. 2015; Chen et al. 2016). Previous studies have shown that limma-based approaches achieve comparable or improved false discovery rate (FDR) control relative to negative binomial methods while providing a flexible linear model suitable for our multifactor experimental design (Law et al. 2014). To control for multiple testing across all pairwise comparisons, global FDR correction was applied. Genes with an FDR below 0.05 were classified as significantly upregulated or downregulated. Using this method, we performed 3 different types of pairwise comparisons: (i) To determine how the trajectory of the MZT changed with heat stress, we performed pairwise comparisons between NC14 and NC9 genes for the nonstress 18 °C control, acute 32 °C heat stress, and chronic 32 °C heat stress conditions. (ii) To determine how the major wave of the MZT at NC14 differs with exposure to heat stress, we examined pairwise comparisons between the acute 32 °C heat stress or chronic 32 °C heat stress genes and nonstress 18 °C control genes at NC14. (iii) To also determine how the minor wave of the MZT at NC9 differs with exposure to heat stress, we repeated the analogous pairwise comparisons between the acute 32 °C heat stress or chronic 32 °C heat stress genes and nonstress 18 °C control genes at NC9.

To ensure that the observed differential expression patterns were not disproportionately driven by only low-abundance genes, mean-average (MA) plots were generated for each MZT comparison under the different experimental conditions. Mean expression values were calculated exclusively from the samples included in the corresponding comparison and plotted as mean log_2_(CPM) against log_2_ fold change (logFC) (Supplementary Fig. 2). These plots indicate that observed transcriptional changes occur for genes with a broad range of expression levels and are not obviously biased by low-abundance genes.

To further reveal meaningful differences in gene expression between conditions, interaction tests were conducted to compare the magnitude and direction of transcriptional changes between matched pairwise contrasts. Specifically, interaction terms were defined to test whether the logFC between NC14 and NC9 at 32 °C, for either acute or chronic heat stress, differed significantly from the corresponding logFC between NC14 and NC9 at 18 °C. This allows us to distinguish between genes that may not be “significant” in 1 pairwise comparison due to hard thresholding of the FDR cutoff (e.g. 0.051). Genes showing significant interaction effects (FDR <0.05) were interpreted as exhibiting stress-dependent modulation of expression during the MZT.

#### Overrepresentation analysis

Overrepresentation analysis was conducted separately for upregulated and downregulated DEGs in each pairwise comparison to identify enriched gene ontology (GO) terms using the PANGEA tool with the “Direct GO Biological Processes” gene ontology set (Hu et al. 2023). The background set included all 7,613 genes that passed the initial filtering criteria.

### Comparison between MZT genes and reference gene sets

To assess the effects of heat stress on zygotic genome activation and maternal transcript clearance during the MZT, DEGs identified between NC9 and NC14 embryos were compared with previously published zygotic and maternal reference gene sets (Supplementary Table 1). Reference zygotic gene sets were obtained from De Renzis et al. (2007), Lott et al. (2011), and Kwasnieski et al. (2019). For the Kwasnieski *et al*. dataset, genes classified as zygotically expressed in both syncytial and cellularized blastoderm embryos were included in the zygotic reference set. Reference maternal genes were obtained from Lott et al. (2011). Gene identifiers from the RNA-seq dataset presented here and the reference gene sets were updated to current FlyBase identifiers using the FlyBase “ID Validator” tool (Öztürk-Çolak et al. 2024), and then matched to determine zygotic and maternal genes under control and heat stress MZT conditions (Supplementary Tables 2 to 4).

For each reference zygotic gene set, the proportion of matched genes identified herein, with direction of regulation (up, down, or not significant), was calculated for each experimental condition (Supplementary Table 5). Upregulated genes from the current dataset were interpreted as evidence of maintained zygotic genome activation, whereas reductions in the number of upregulated genes and increases in downregulated and/or not significant genes were interpreted as evidence of deviation from the MZT. To evaluate the extent to which heat stress altered overall regulatory patterns, χ^2^ tests on 2 × 3 contingency tables (with significance threshold *P* < 0.05) were applied to compare the distributions of upregulated, downregulated, and not significant genes between control nonstress and heat stress conditions (Supplementary Table 5). To identify specific regulatory categories contributing to significant differences, category-specific χ^2^ tests using 2 × 2 contingency tables (with an adjusted *P* < 0.05 significance threshold) were applied, comparing the proportion of genes in each category with that of all remaining genes within the reference gene set (Supplementary Table 6). The *P* values from category-specific analyses were adjusted using the Benjamini–Hochberg method.

For the maternal gene set analysis, 5,279 genes in our RNA-seq dataset matched the maternal gene set defined by Lott et al. (2011) (Supplementary Tables 3 and 7). Of these, 1,956 genes were downregulated during the control MZT (18 °C) and were subsequently used as the reference set. The proportion of these genes that were upregulated, downregulated, or not significantly changed was then determined for the acute and chronic heat stress MZT conditions (Supplementary Table 8). Downregulated genes from the current dataset were interpreted as representing normal maternal transcript clearance during the MZT. Alternatively, loss of downregulation under heat stress was interpreted as a disruption of maternal mRNA clearance (Supplementary Table 8). To evaluate the extent to which heat stress altered maternal transcript clearance, χ^2^ tests on 2 × 3 contingency tables (with significance threshold *P* < 0.05) were applied to compare the distributions of upregulated, downregulated, or not significant genes between heat stress conditions (Supplementary Table 8).

To determine the proportions of zygotic genes that are upregulated and downregulated during acute and chronic heat stress at NC14, DEGs identified in heat-stressed embryos (32 °C versus 18 °C) were matched to the genes defined as zygotic by all 3 reference zygotic gene sets (De Renzis et al. 2007; Lott et al. 2011; Kwasnieski et al. 2019) (Supplementary Tables 9 to 16). Similarly, to determine whether acute and chronic heat stress at NC14 and chronic heat stress at NC9 preferentially affects maternal or zygotic genes, DEGs identified in heat-stressed embryos (32 °C versus 18 °C) were matched to the gene classifications reported by Lott et al. (2011) and assigned to 1 of the 3 categories: maternal, maternal + zygotic, or zygotic (Supplementary Tables 10, 12, and 15 to 20). The percentage of upregulated or downregulated genes belonging to each gene class was then calculated (Supplementary Tables 17, 18, and 20).

### Data visualization

All transcriptomic visualizations were generated in R (v4.4.0). Volcano plots were created using ggplot2 and ggrepel from differential expression results obtained via the limma-trend pipeline. Genes were categorized as upregulated (log_2_FC > 0, FDR < 0.05), downregulated (log_2_FC < 0, FDR < 0.05), or not significant. The top 15 most significant genes in each direction (based on FDR) were labeled on the plots. Each volcano plot displayed log_2_ fold change vs −log_10_(FDR) with significance thresholds (FDR = 0.05) indicated by dotted lines.

UpSet plots were generated using the UpSetR and dplyr packages to visualize overlaps among gene sets significantly up- or downregulated (FDR < 0.05) across contrasts.

Enrichment barplots were generated from PANGEA GO enrichment results using ggplot2, dplyr, and readr. Up- and downregulated GO tables were ranked by −log_10_(*P*) values, and the top enriched terms were displayed as horizontal barplots. Final figure panels were made in Adobe Illustrator 2021.

### Real-time qPCR

To validate the RNA-seq data, qPCR analysis of 7 HSP genes was performed in NC14 embryos (Hsp83, Hsp70, Hsp68, Hsp26, Hsp23, Hsc70-4, and DNAJ-1). RNA was isolated from 10 to 20 hand-selected embryos at NC14 as described above for RNA-seq, with 3 biological replicates performed for each experimental condition. RNA concentration and quality were assessed using a NanoDrop 2000c Spectrophotometer (Thermo Fisher Scientific, Waltham, USA). Per sample, a total of 300 ng of extracted RNA was used to perform reverse transcription with iScript Select cDNA Synthesis Kit (Catalog# 1708897, Bio-Rad Laboratories), and a 1:5 cDNA dilution was prepared with Molecular Biology Grade Water (Catalog# 46-000-CM, Corning, Manassas, USA). Real-time qPCR was performed on Applied Biosystems StepOnePlus Real-Time PCR system utilizing TaqMan Fast Advanced Master Mix (Catalog# 4444557, Applied Biosystems, Thermo Fisher Scientific, Vilnius, Lithuania) and predesigned TaqMan Gene Expression Assays (Catalog# 4448892, 20×; Applied Biosystems, Thermo Fisher Scientific, Waltham, USA) for Hsp68 (Assay ID: Dm02151262_s1), Hsp26 (Assay ID: Dm01822452_s1), Hsp23 (Assay ID: Dm01822473_s1), Hsp83 (Assay ID: Dm02362342_s1), Hsc70-4 (Assay ID: Dm02153823_s1), and DnaJ-1 (Assay ID: Dm01832926_s1), and a custom Assay was used for Hsp70. We selected roadblock (Robl) as a housekeeping gene (Catalog# 4351372, Assay ID: Dm01821522_g1) to normalize the Ct values of each target gene. This selection was due to the stable mRNA expression levels of the Robl gene for all conditions for NC14 embryos and its prior evaluation by other researchers (Ling and Salvaterra 2011; Lü et al. 2018). The relative mRNA expression level of each target gene was calculated using the 2^-ΔΔCt method (Schmittgen and Livak 2008).

### Comparative validation of qPCR and RNA-seq expression profiles

To validate RNA-seq results in NC14 embryos with qPCR data, gene expression differences between experimental conditions were analyzed using one-way ANOVA on both qPCR-derived normalized 2^-ΔΔCt and RNA-seq logCPM values. When differences found were significant, post hoc Tukey's honestly significant difference (HSD) test was applied for all pairwise comparisons (acute 32 °C heat stress versus nonstress 18 °C control; chronic 32 °C heat stress versus nonstress 18 °C control; and acute 32 °C versus chronic 32 °C heat stress). This analysis was performed consistently for all genes without prefiltering for normality or variance equality. Our qPCR analysis of 7 HSP genes under heat stress confirmed concordant expression patterns in NC14 embryos with a reproducibility rate of 92.8% (ie consistent direction of regulation was detected for 7 genes in 13 out of 14 events; Supplementary Fig. 3).

Statistical analyses and plot generation were performed in R (v4.4.0) utilizing the packages readxl, dplyr, tidyr, ggplot2, ggpubr, rstatix, stringr, and grid. Bar plots showing mean ± SEM were generated using ggplot2.

## Results

### A core MZT persists despite heat stress

We considered that the pairwise comparison between the major and minor waves of zygotic genome activation (i.e. NC14 versus NC9) for a given experimental condition represents the genes that are differentially expressed during the MZT for that condition (see Fig. 1a for experimental design). Corresponding survival data per condition were also determined (Fig. 1b). We found that for the nonstress 18 °C control embryos, 3,126 genes were upregulated and 2,643 downregulated during the MZT (FDR < 0.05; Fig. 1c). Enrichment analysis aligned with expectations that upregulated genes during the MZT should be related to biological processes involved in gene regulation (e.g. transcription, splicing, and translation) and tissue and organ development (e.g. morphogenesis and axon guidance) (Fig. 1d; Lécuyer et al. 2007; Chen et al. 2013; Hamm and Harrison 2018; Brantley and Di Talia 2024; Kojima et al. 2025; Pérez-Mojica et al. 2025). Among the downregulated genes, DNA replication was the top biological process, consistent with the cessation of the rapid embryonic cleavage cycles at the major wave of the MZT at NC14 (Brantley and Di Talia 2024). Female meiotic chromosome segregation genes were also downregulated, demonstrating degradation of maternally contributed gene products. In addition, processes related to lipid metabolism were downregulated in line with recent reports that metabolic reprogramming accompanies the MZT (Kilwein et al. 2023; Pérez-Mojica et al. 2025). Comparison between MZT genes identified here and reference zygotic gene sets (i.e. zygotically expressed) for nonstressed embryos from De Renzis et al. (2007), Lott et al. (2011), and Kwasnieski et al. (2019) showed 98.2%, 44.1%, and 65.9% overlap, respectively (Supplementary Tables 2 to 5). For a maternal gene set reported in Lott et al. (2011), we found 96.7% overlap with our data (Supplementary Table 7). Thus, our analysis for nonstressed embryos is able to detect genes associated with an MZT that is manifest across diverse genetic backgrounds and methodologies (Supplementary Tables 1, 5, and 7) and correlates with high embryo survival (Fig. 1b).

For the acute 32 °C heat stress condition, slightly more genes were differentially expressed with 3,175 genes upregulated and 2,730 downregulated during the MZT (FDR < 0.05; Fig. 1e). Nonetheless, the most significant enrichment terms were largely equivalent to the nonstress 18 °C control embryos for both up- and downregulated genes (Fig. 1f). Similarly, comparison between upregulated MZT genes identified for acutely stressed embryos and matching genes from reference zygotic gene sets for nonstressed embryos retained ≥78.7% overlap, with no statistically significant difference for 2 of the 3 reference gene sets (Fig. 2a; Supplementary Tables 5 and 6). This suggests that the core MZT is largely preserved despite acute heat stress: an interpretation supported by high larval hatching rates at this condition (Fig. 1b).

**Fig. 2.**
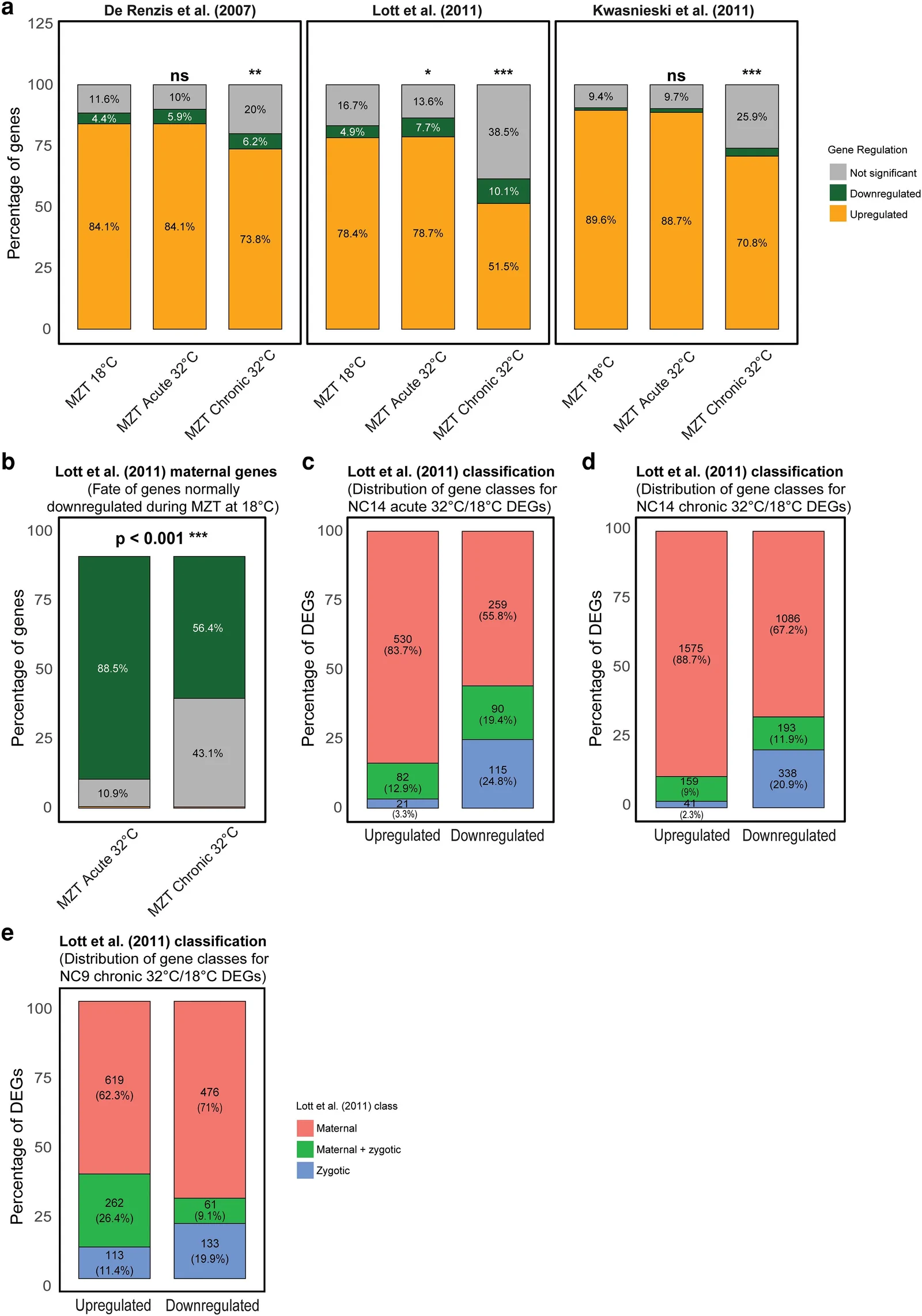
Comparison of MZT in heat-stressed embryos with reference maternal and zygotic gene lists. a) Stacked barplots showing the proportion of upregulated, downregulated, and not significant genes within previously defined zygotic gene sets, as indicated, across nonstress 18 °C control (MZT 18 °C), acute 32 °C heat stress (MZT acute 32 °C), and chronic 32 °C heat stress (MZT chronic 32 °C) conditions. Stacked bars are color coded by regulation category (gray, top segment = not significant; green, middle segment = downregulated; orange, bottom segment = upregulated). b) Stacked barplots showing the proportion of genes classified as maternal that are upregulated, downregulated, or not significantly changed during the MZT under acute 32 °C heat stress and chronic 32 °C heat stress conditions. Stacked bars are color coded by regulation category (green, top segment = downregulated; gray, middle segment = not significant; orange, bottom segment = upregulated). Percentage labels are shown only for bar segments representing ≥4% of genes. Significance thresholds: ns, not significant; * adjusted *P* < 0.05; ** adjusted *P* < 0.01; *** adjusted *P* < 0.001. c, d, and e) For within NC14 (c and d) or within NC9 (e), stacked barplots showing the distribution of upregulated and downregulated genes identified for acute 32 °C heat stress (c), and chronic 32 °C heat stress (d and e) conditions compared to nonstress 18 °C controls across classes of genes previously defined by Lott et al. (2011) in early embryos (pink, top segment = maternal; green, middle segment = maternal + zygotic; blue, bottom segment = zygotic). Values within bars indicate the number and percentage of overlapping significant DEGs belonging to each class of reference genes.

For the more intense chronic 32 °C heat stress, many fewer genes changed during the MZT, with only 1,983 genes upregulated and 1,906 downregulated (FDR < 0.05; Fig. 1g). While this was a more dramatic disruption of the MZT than observed for acute heat stress, the enrichment analysis still showed that many of the expected biological processes persisted in embryos after chronic heat stress exposure, particularly when looking at the upregulated genes (e.g. transcription, splicing, translation, and morphogenesis; Fig. 1h). Among the downregulated genes, some expected biological processes also remained (e.g. DNA replication). But some indicators of heat stress emerged that were unique to the chronic heat stress condition, including terms related to proteosome assembly, starvation, and oxidation–reduction processes (Fig. 1h; Pessa et al. 2024; Genuth and Dillin 2025). Comparison between upregulated MZT genes identified for the chronically heat stressed embryos and matching genes from reference zygotic gene sets for nonstressed embryos showed that a majority of genes still overlapped (≥51.5%; Fig. 2a; Supplementary Tables 5 and 6), again indicating some maintenance of the MZT. However, the difference between the MZT for chronic 32 °C heat stress and all three reference gene sets was statistically significant (Fig. 2a; Supplementary Tables 5 and 6). Thus, a core MZT profile persists for chronically heat-stressed embryos, but with obvious differences from either nonstressed or acutely stressed embryos, and correlating with poorest embryo survival, as measured by the larval hatching assay (Fig. 1b).

### Deviations from the core MZT under chronic heat stress involve both zygotic genome activation and maternal transcript clearance

Based on our observation that fewer genes are upregulated or downregulated during the MZT for chronically heat-stressed embryos (Fig. 1g), we hypothesized that both zygotic genome activation and maternal transcript clearance are suppressed in these embryos. Comparison between genes defined as zygotic in the three published reference datasets (De Renzis et al. 2007; Lott et al. 2011; Kwasnieski et al. 2019) and matching genes upregulated during the MZT under chronic heat stress consistently showed that a significantly reduced percentage of zygotic genes are activated in chronically heat-stressed embryos (Fig. 2a; Supplementary Tables 5 and 6). Comparison of maternal genes defined by Lott et al. (2011) that were downregulated during the control MZT (18 °C) similarly showed that a reduced percentage of maternal genes are downregulated in chronically heat-stressed embryos (Fig. 2b; Supplementary Table 8). Thus, we suggest that, while a core MZT can be maintained with heat stress, the more intense the heat stress, the greater the compromise to both activation of the zygotic genome and decay of maternal transcripts.

### Deviations from the core MZT are distinct for the acute versus chronic heat stress conditions with greater developmental impact revealed as stress intensifies

To further assess the extent to which the MZT differs from normal in heat-stressed embryos, we used the individual pairwise comparisons and interaction tests, which identify significant differences between individual fold changes, to classify genes into 3 categories: (i) common responses, (ii) unique responses, or (iii) borderline responses (Fig. 3a and b). Common responses were genes with significant pairwise changes in the same direction and a nonsignificant interaction. Unique responses were genes significant in only one pairwise comparison with a significant interaction, or significant in opposite directions with a significant interaction. Borderline cases were genes that were significant in only one pairwise comparison but had a nonsignificant interaction, or genes with significant pairwise changes in the same direction but a significant interaction indicating a difference in effect size. Because these latter cases were not clearly common or unique, we did not consider them further. When comparing the MZT of acute 32 °C heat-stressed and 18 °C nonstressed control embryos, we found the majority of genes are in common (2,792 upregulated and 2,244 downregulated; Fig. 3a). For the MZT of chronic 32 °C heat-stressed embryos, a majority of genes remain in common with 18 °C control embryos (1,638 upregulated and 1,383 downregulated; Fig. 3b). Among the unique genes, there were relatively few genes for each heat stress condition (for acute stress, 73 (60 + 13) upregulated and 90 (85 + 5) downregulated genes; for chronic stress, 59 (49 + 10) upregulated and 131 (124 + 7) downregulated genes; Fig. 3a and b). These findings support the idea that the MZT is fairly robust across temperature perturbations, and the biological processes associated with the common genes for both acute and chronic heat stress align with this interpretation (e.g. transcription, splicing, translation, and morphogenesis for upregulated genes and DNA replication for downregulated genes; Fig. 3c and d).

**Fig. 3.**
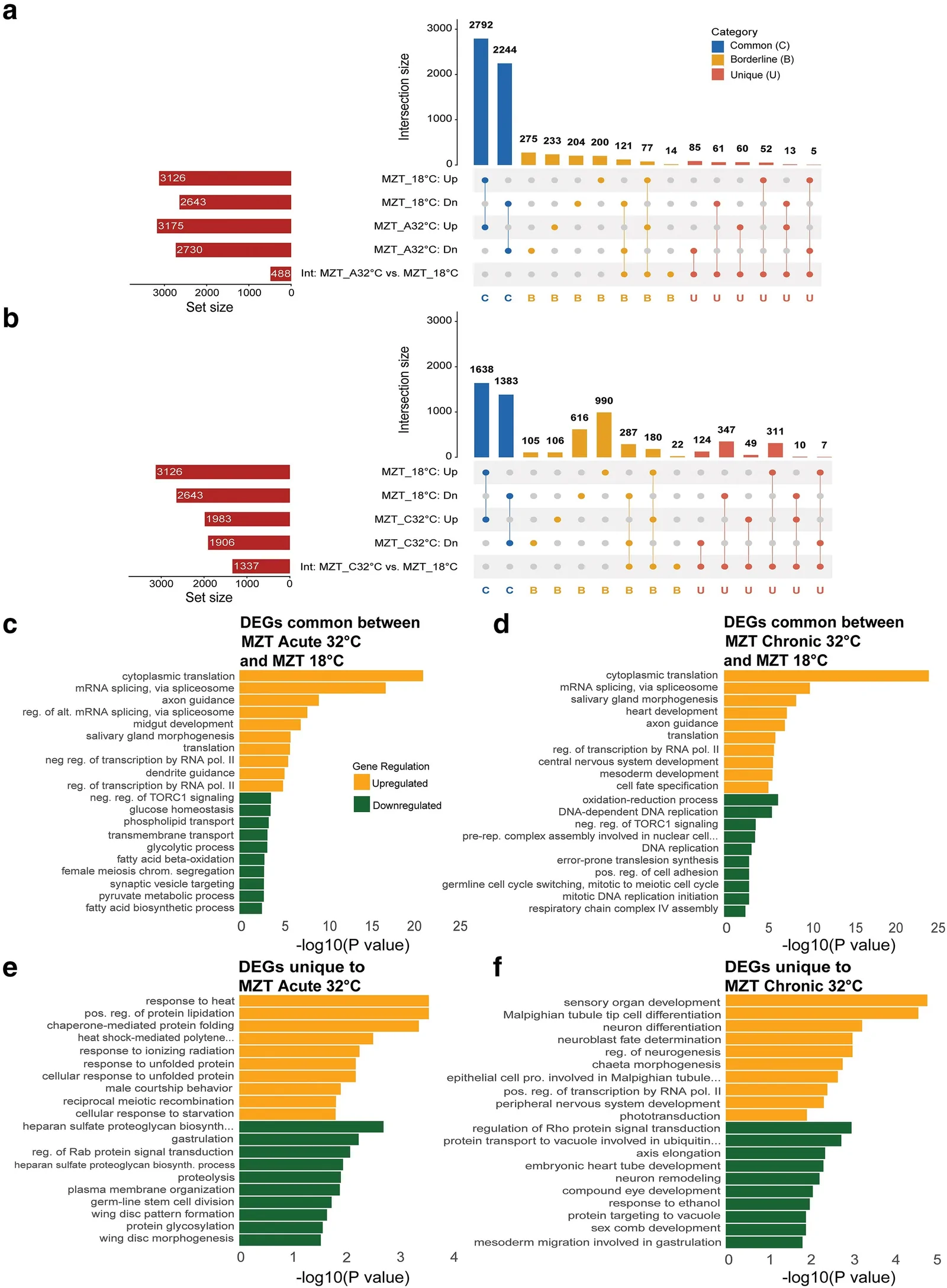
Pairwise and interaction (Int) tests to identify genes in common or unique to the MZT when embryos are exposed to acute or chronic heat stress. a) UpSet plot showing intersections of upregulated (Up) and downregulated (Dn) MZT genes between control 18 °C (MZT_18 °C) and acute 32 °C heat stress (MZT_A32 °C) conditions, together with genes showing a significant interaction between conditions . b) UpSet plot showing intersections of Up and Dn MZT genes between MZT_18 °C and chronic 32 °C heat stress (MZT_C32 °C), including interaction-significant sets. a and b) Numbers above the bars in the main plot indicate the number of genes per set; vertical lines show intersecting sets; dark red bars on the left show the number of significant genes in each comparison. The letters below each comparison show which category each set was classified as with C = common (blue), U = unique (salmon red), and B = borderline (orange). c-f) GO Biological Process enrichment for acute (c and e) or chronic (d and f) common or unique genes, as indicated. The top 10 terms are ranked by −log_10_(*P* value) for upregulated (orange, top bars) and downregulated (green, bottom bars) gene sets. Abbreviations: reg., regulation; pol., polymerase; pos., positive; alt., alternative; neg., negative; biosynth., biosynthetic; prerep., prereplicative; protein transport to vacuole involved in ubiquitin…, protein transport to vacuole involved in ubiquitin-dependent protein catabolic process via the multivesicular body sorting pathway; heat shock-mediated polytene…, heat shock-mediated polytene chromosome puffing; heparan sulfate proteoglycan biosynth…, heparan sulfate proteoglycan biosynthetic process; prerep. complex assembly involved in nuclear cell…, prerep. complex assembly involved in nuclear cell cycle DNA replication; epithelial cell pro. involved in Malpighian tubule…, epithelial cell proliferation involved in Malpighian tubule morphogenesis.

However, the unique genes also highlight important deviations. Comparing the MZT of acute 32 °C heat stress to the nonstress 18 °C control, the unique gene set shows that HSR is induced with biological processes such as response to heat and response to unfolded protein identified for upregulated genes and proteolysis identified for downregulated genes (Fig. 3e). These are expected hallmarks of HSR (Pessa et al. 2024; Genuth and Dillin 2025). A few biological processes relevant to development are seen, particularly among the downregulated genes. In contrast, for the unique genes for the MZT in chronic 32 °C heat stress compared to the nonstress 18 °C control, almost all terms are associated with development for both upregulated and downregulated genes, with some possible signs of heat stress, including protein catabolic process (Fig. 3f). Thus, chronic stress results in fewer genes being differentially expressed during the MZT (Fig. 1g) and more developmental genes showing misexpression. Despite some maintenance of an MZT in the chronic heat stress condition, the reduced survival of these embryos suggests that development is meaningfully disrupted (Fig. 1b).

### Deviations from the core MZT are largely associated with maternal genes for both acute and chronic stress

As another way to scrutinize the differences that accompany the MZT in heat-stressed embryos, we also considered how transcriptomes are altered at the distinct timepoints of either the major or minor wave of zygotic genome activation. For the major wave, we first compared NC14 in acute 32 °C heat stress to NC14 in nonstress 18 °C control embryos. We found 683 upregulated genes and 500 downregulated (FDR < 0.05; Fig. 4a), with enrichment analysis showing that HSR was induced in the acute stress condition. Specifically, for the upregulated genes, the 10 most significant biological processes were all related to HSR (e.g. response to heat, protein refolding, and chaperone-mediated protein folding; Fig. 4b).

**Fig. 4.**
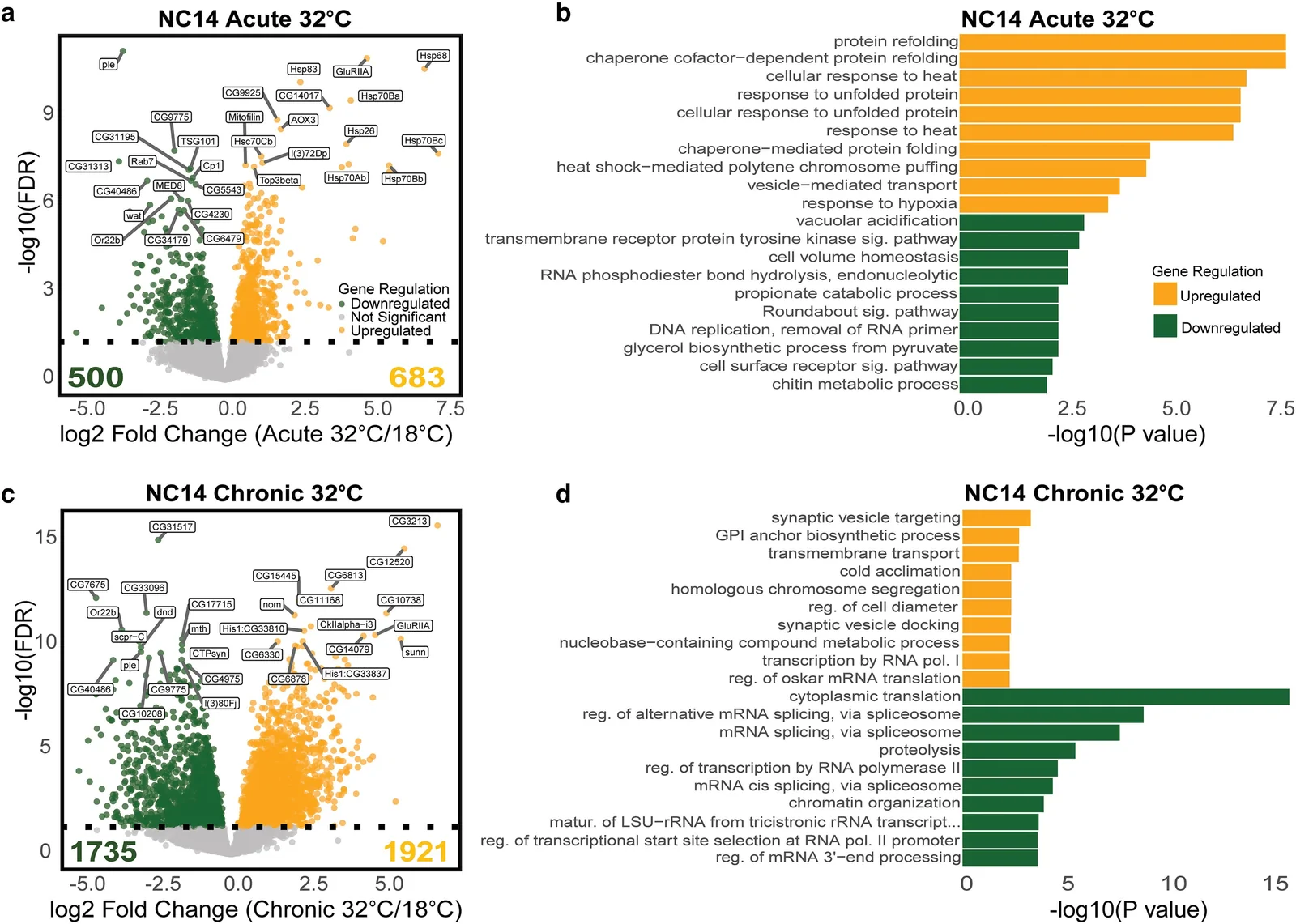
DGE for NC14 embryos exposed to acute or chronic heat stress. a and c) Volcano plots showing differential expression between NC14 embryos exposed to acute 32 °C heat stress (a) or NC14 embryos exposed to chronic 32 °C heat stress (c) compared to nonstress 18 °C controls (orange, upper right wing = upregulated; green, upper left wing = downregulated; *x* axes show logFC, and *y* axes show −log_10_(FDR)). The top 15 upregulated and downregulated genes are labeled. b and d) GO Biological Process enrichment for NC14 upregulated and downregulated genes under acute 32 °C heat stress (b) or chronic 32 °C heat stress (d). The top 10 terms are ranked by −log_10_(*P* value) for upregulated (orange, top bars) and downregulated (green, bottom bars) gene sets. Abbreviations: sig., signaling; reg., regulation; pol., polymerase; matur., maturation; matur. of LSU-rRNA from tricistronic rRNA transcript…, matur. of LSU-rRNA from tricistronic rRNA transcript (SSU-rRNA, 5.8S rRNA, LSU-rRNA).

However, we expected that not all DEGs at NC14 for acute 32 °C heat stress would be associated with stress-related functions, and some would instead be involved in development. To test this, we matched the 1,183 DEGs (683 + 500) to the published reference zygotic and maternal gene sets (Supplementary Tables 9 to 12; De Renzis et al. 2007; Lott et al. 2011; Kwasnieski et al. 2019). We found a low number of matching zygotic genes with only 35, 136, and 116 overlapping with De Renzis et al. (2007), Lott et al. (2011), and Kwasnieski et al. (2019), respectively (Supplementary Table 12). This aligns with our earlier observation that zygotic genome activation persists in the acute heat stress condition (Fig. 1e). In contrast, a large number of DEGs (*n* = 789) matched the Lott et al. (2011) purely maternal gene set (Supplementary Tables 10, 12, and 17). These genes were defined as maternally provided and downregulated during the MZT in nonstressed embryos (Lott et al. 2011) yet were represented by both up- and downregulated genes at NC14 in the acute 32 °C heat stress embryos (Fig. 2c; Supplementary Tables 12 and 17). Thus, deviation from the core MZT following acute heat stress is not only due to the induction of stress response but may also arise when maternal transcript clearance is aberrantly suppressed or stimulated for specific maternal mRNAs.

We next looked at the major wave of zygotic genome activation for the chronic 32 °C heat stress condition, where 1,921 genes were upregulated and 1,735 downregulated at NC14 compared to the nonstress 18 °C control (FDR < 0.05; Fig. 4c). For chronic stress, heat stress response was not immediately revealed by enrichment analysis for the upregulated genes (Fig. 4d), although levels of some HSPs were increased (Supplementary Fig. 3). However, the downregulated genes were associated with biological processes that are expected to be suppressed or modified by heat stress, including translation, mRNA processing, chromatin organization, and proteolysis (Fig. 4d; Pessa et al. 2024; Genuth and Dillin 2025). Perhaps this represents some adapted physiological state for the chronically stressed embryos.

Again, we posited that a portion of the DEGs at NC14 for chronic 32 °C heat stress would be associated with development rather than stress response functions. We matched the 3,656 DEGs (1,921 + 1,735) to the reference zygotic and maternal gene sets (De Renzis et al. 2007; Lott et al. 2011; Kwasnieski et al. 2019). We found a higher number of matching zygotic genes differentially expressed in chronic stress than we had for acute stress, with 128, 379, and 285 genes overlapping with De Renzis et al. (2007), Lott et al. (2011), and Kwasnieski et al. (2019), respectively (Supplementary Tables 13 to 16). However, as was the case for acute heat stress, the highest matching was observed between the DEGs at NC14 in chronically stressed embryos and the Lott et al. (2011) purely maternal gene set, with 2,643 genes overlapping (Supplementary Tables 14, 16, and 18). Matched genes again showed both up- and downregulation at NC14 in the chronic 32 °C heat stress embryos (Fig. 2d; Supplementary Tables 16 and 18). Thus, deviation from the core MZT following chronic heat stress seems to comprise both modulation of or adaptation to heat stress and misregulation of maternal mRNA clearance.

To examine the minor wave of zygotic genome activation, we compared NC9 in acute 32 °C heat stress to NC9 in nonstress 18 °C control embryos. Few genes changed for the acute stress condition, with 71 genes upregulated and 118 genes downregulated (FDR < 0.05; Fig. 5a and b). The low number of genes may reflect that at NC9, zygotic transcription is just beginning, and maternal transcripts are likely to dominate for both the heat stress and nonstress conditions (Tadros and Lipshitz 2009; Hamm and Harrison 2018; Kojima et al. 2025).

**Fig. 5.**
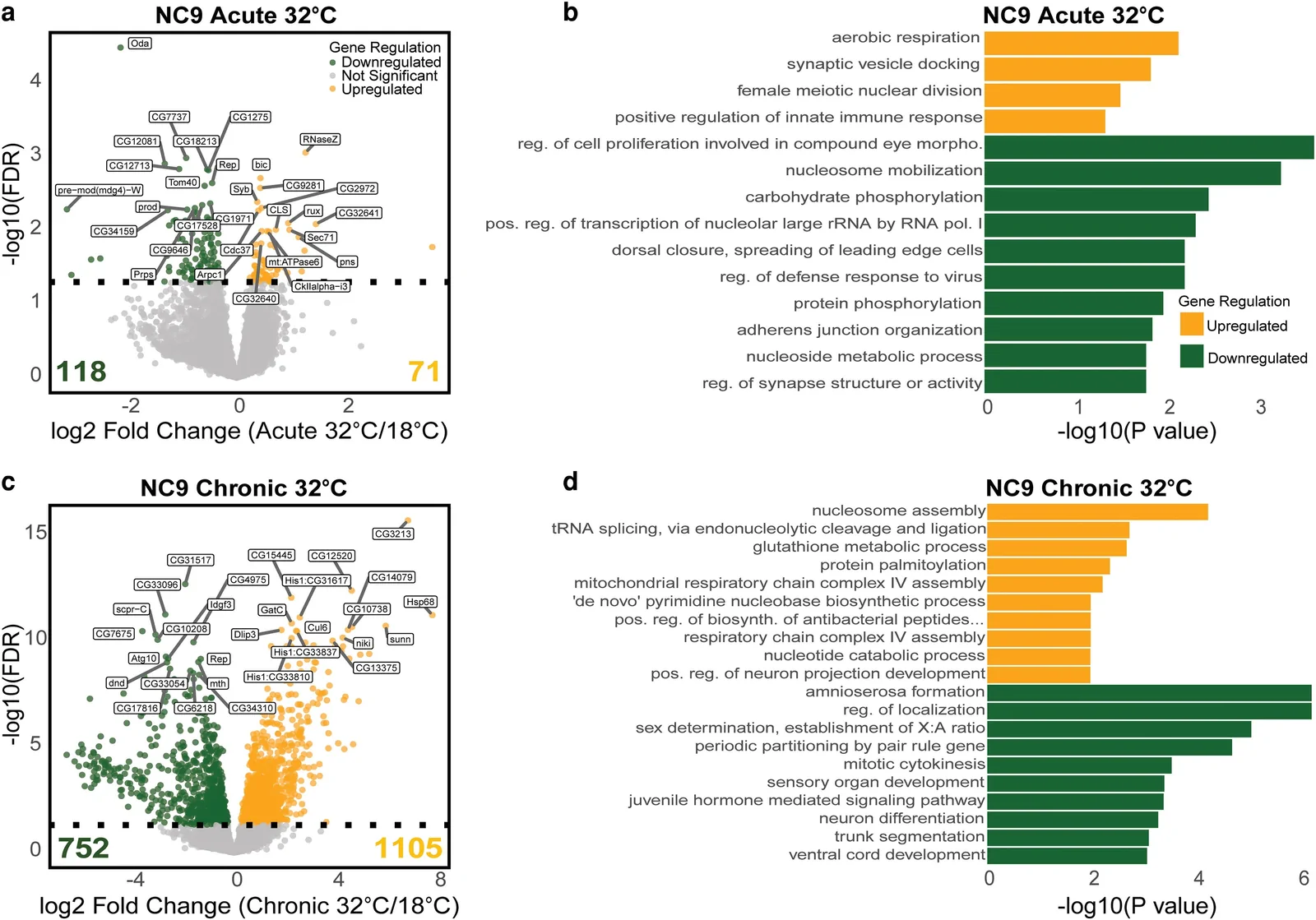
DGE for NC9 embryos exposed to acute or chronic heat stress. a and c) Volcano plots showing differential expression between NC9 embryos exposed to acute 32 °C heat stress (a) or NC9 embryos exposed to chronic 32 °C heat stress (c) compared to nonstress 18 °C controls (orange, upper right wing = upregulated; green, upper left wing = downregulated; *x* axes show logFC, and *y* axes show −log_10_(FDR)). The top 15 upregulated and downregulated genes are labeled. b and d) GO Biological Process enrichment for NC9 upregulated and downregulated genes under acute 32 °C heat stress (b) or chronic 32 °C heat stress (d). The top 10 terms are ranked by −log_10_(*P* value) for upregulated (orange, top bars) and downregulated (green, bottom bars) gene sets. For (b), only the top four bars represent the upregulated gene set. Abbreviations: biosynth., biosynthetic process; reg., regulation; pos., positive; pol., polymerase; pos. reg. of biosynth. of antibacterial peptides…, pos. reg. of biosynth. process of antibacterial peptides active against Gram-negative bacteria.

That being the case, it is intriguing that a much larger number of genes are differentially expressed at NC9 following chronic 32 °C heat stress exposure, with 1,105 genes upregulated and 752 genes downregulated (FDR < 0.05; Fig. 5c). In our experimental scheme, the initial exposure to chronic heat stress occurs when oocytes are still developing in mothers. So, we suggest that the larger number of DEGs at NC9 for embryos after chronic stress reflects a change in the maternally loaded gene products. Indeed, of the 1,664 DEGs that could be assigned a class based on Lott et al. (2011), 1,095 (65.8%) genes show overlap with the purely maternal gene set, with matching genes showing both up- and downregulation at NC9 in the chronic 32 °C heat stress embryos (Fig. 2e; Supplementary Tables 19 and 20). Upregulated genes are associated with terms such as assembly of nucleosomes and mitochondrial respiratory chain IV complex, positive regulation of biosynthetic processes, and glutathione metabolic process (Fig. 5d), indicating a potentially distinct physiology compared to the nonstress 18 °C control embryos. For the downregulated genes, the most significantly enriched biological processes are all related to development (e.g. segmentation, morphogenesis, and sex determination; Fig. 5d). These results suggest that embryos in the chronic heat stress condition not only deviate from the trajectory of the MZT in detectable ways (i.e. fewer genes change and some developmental genes are misexpressed), but they also initiate their MZT from a different baseline compared to nonstressed embryos.

## Discussion

Our data shows that a resilient core MZT persists across the 32 °C heat stress exposures tested here. This core MZT is characterized by activation of well-defined MZT processes, including transcription, splicing, translation, patterning, and morphogenesis (Hamm and Harrison 2018; Riemondy et al. 2023; Kojima et al. 2025), and can support the survival of embryos (Fig. 1b). Notably, for acute stress, the activation of HSPs appears to act additively, not antagonistically, with zygotic genome activation during the MZT (Fig. 3e). A core MZT is also maintained for chronic heat stress; however, many fewer genes change than predicted by the nonstress condition (compare Fig. 1c and g). Genes that are uniquely modified in the chronic heat stress condition are strongly biased toward development (Fig. 3f), suggesting that embryogenesis will be more severely impacted. Furthermore, the NC9 transcriptome for chronically heat-stressed embryos already diverges significantly from the nonstressed embryos, likely reflecting an altered maternal contribution of gene products and different baseline for initiation of the MZT. Overall, the seemingly more severe disruptions to the MZT in the chronic stress condition track with its reduced survival outcomes compared to both the nonstress control and acute heat stress conditions. We conclude that the MZT is resilient but becomes increasingly imperiled as the level of stress intensifies.

One perhaps surprising result was that for both the acute and chronic heat stress MZTs, comparison with reference gene sets (De Renzis et al. 2007; Lott et al. 2011; Kwasnieski et al. 2019) revealed that it may be the program of maternal transcript handling and clearance that is more profoundly compromised than zygotic genome activation. To a large extent, it appears that misregulation of maternally provided mRNAs may generally account for the deviations seen for DEGs during the MZT in heat-stressed embryos. This finding aligns with the very well-established robustness known to characterize zygotic transcription (von Dassow et al. 2000; Manu et al. 2009; Frankel et al. 2010; Perry et al. 2010) and may also partially derive from the simple preponderance of maternal transcripts in the early embryos (Lott et al. 2011).

It should be noted that induction of a response to heat stress in these early *Drosophila* embryos cannot be immediately equated with HSR in adult cell types. Previous studies have shown that HSP inducibility is developmentally delayed in diverse early embryos (Roccheri et al. 1982; Morange et al. 1984; Heikkila et al. 1985; Arrigo and Tanguay 1991; Edwards et al. 1997), and in the fly, the inducing transcription factor HSF only gains the capacity for nuclear import at NC13 (Wang and Lindquist 1998). So, while we and others report that embryos are responsive to heat stress, the molecular and cellular mechanisms are expected to diverge in detail from canonical HSR. Indeed, this is why we undertook our experiments: to define what heat stress response does look like in an early embryo at the specific temperatures relevant to our prior work on the actin stress response (Figard et al. 2019). We clearly see that the syncytial-stage fly embryo is competent to upregulate transcription of several HSPs and other chaperones, including Hsp 23, 26, 68, 70, and 83; and we find that this HSP upregulation occurs at a greater magnitude in the acute rather than chronic heat stress condition, perhaps indicative of some adaptation to the prolonged stress.

We present our dataset with the intention that it will provide other researchers a resource to identify molecular pathways and processes that contribute to embryonic outcomes upon heat stress exposure. Using this dataset, one could consider both drivers of heat stress response as well as consequences of the stress response, either protective or maladaptive. In addition, this dataset serves as a systemic readout of heat stress response in early fly embryos at a temperature that is known to permit but also challenge survival (Kuntz and Eisen 2014; Chen et al. 2015; Schou et al. 2017; Chong et al. 2018; Crapse et al. 2021; Falahati et al. 2021; Kale et al. 2026; Gibbs et al. 2025). While scores of earlier seminal studies shed light on the impacts of short heat shocks at lethal temperatures for flies (37 to 42 °C), showing global reductions in transcription and translation and activation of certain HSPs (Graziosi et al. 1980; Findly and Pederson 1981; Lindquist 1981; Vazquez et al. 1993), there was no clear prediction regarding the extent of HSR induced by our more moderate heat stress exposures, even though these conditions are plausibly encountered by flies in native habitats (Feder 1997). In fact, it has been questioned whether early embryos will mount an HSR at all, because it would be too perturbative to gene expression programs, such as the MZT (Hamdoun and Epel 2007; Mikucki et al. 2024; Pessa et al. 2024). However, our dataset now helps to clarify expectations regarding both the capacity of early embryos to respond to heat stress and the susceptibility of the MZT to increasingly severe environmental perturbation.

## Supplementary Material

jkag203_Supplementary_Data

## Data Availability

The raw RNA-seq datasets for this study can be found in the US National Institutes of Health/National Center for Biotechnology Information/Sequence Reads Archive via accession number: PRJNA1196936. The processed datasets, metadata, and analysis scripts can be found in the Illinois Data Bank at DOI: https://doi.org/10.13012/B2IDB-3845814_V1. Supplemental material available at *G3* online.
