# Supplementary material for: Transcriptomic profiles of the maternal-to-zygotic transition for Drosophila embryos exposed to acute and chronic heat stress
Source: G3 (Bethesda). 2026 Aug 13;16(10):jkag203. doi: 10.1093/g3journal/jkag203 (PMC13643814; doi:10.1093/g3journal/jkag203)
Supplement: Supplementary file 1 [file jkag203_supplementary_data.zip › Supplementaryfigurelegends.docx]

# Supplementary figure legends

**Figure S1. TMM normalization factors for RNA-seq libraries.** Bar plot showing TMM normalization factors calculated for all RNA-seq libraries after filtering genes with CPM ≥ 1 in at least three samples. TMM normalization factors were calculated using the edgeR package to account for differences in library composition among samples. Each bar represents the normalization factor for an individual library, and the horizontal line indicates a value of 1.0 (no scaling). Values close to 1.0 across samples indicate minimal differences in library composition and suggest that only modest normalization (scaling) adjustments were required prior to differential expression analysis.

**Figure S2. MA plots for MZT gene expression analyses.** a-c) MA plots showing DGE between NC14 and NC9 embryos under (a) control 18°C, (b) acute 32°C heat stress, and (c) chronic 32°C heat stress conditions. The x-axes show mean log2(CPM) calculated from samples included in each comparison, and the y-axes show log2 fold change (NC14/NC9).

Genes with FDR < 0.05 were considered significant (orange, upper cluster = upregulated; green, lower cluster = downregulated; gray, central cluster = not significant). DEGs are distributed across a wide range of expression levels, indicating that the observed expression changes are not predominantly driven by low-abundance transcripts.

**Figure S3. qPCR validation of RNA-seq results using heat stress gene expression changes in NC14 embryos for each experimental condition.** (a) Bar plots showing qPCR-derived 2^-ΔΔCt values for 7 HSP genes (Hsp83, Hsp70, Hsp68, Hsp26, Hsp23, Hsc70-4, DNAJ-1) in NC14 embryos. The x-axis shows gene names, and the y-axis shows normalized relative expression (2^-ΔΔCt). (b) Bar plots showing corresponding RNA-seq expression levels for the same HSP genes. The x-axis shows heat stress genes, and the y-axis shows logCPM values. (a and b) Bars are color-coded by condition from left to right (brown = nonstress 18°C control, blue = acute 32°C heat stress, orange = chronic 32°C heat stress). Statistical analysis was performed using one-way ANOVA, followed by Tukey’s HSD for pairwise comparisons (nonstress 18°C control versus acute 32°C heat stress, nonstress 18°C control versus chronic 32°C heat stress, acute 32°C heat stress versus chronic 32°C heat stress). Significance thresholds are indicated as: (*) p ≤ 0.05, (**) p ≤ 0.01, (***) p ≤ 0.001, (^) p < 0.10, (ns) = not significant.
